# CXCL5 induces tumor angiogenesis via enhancing the expression of FOXD1 mediated by the AKT/NF-κB pathway in colorectal cancer

**DOI:** 10.1038/s41419-019-1431-6

**Published:** 2019-02-21

**Authors:** Chun Chen, Zhuo-Qing Xu, Ya-Ping Zong, Bao-Chi Ou, Xiao-Hui Shen, Hao Feng, Min-Hua Zheng, Jing-Kun Zhao, Ai-Guo Lu

**Affiliations:** 1 Shanghai Minimally Invasive Surgery Center, Ruijin Hospital, Shanghai Jiaotong University School of Medicine, Shanghai, China; 2 Gastroenterology Surgery Department, Ruijin Hospital, Shanghai Jiaotong University School of Medicine, Shanghai, China; 3 Shanghai Institute of Digestive Surgery, Shanghai, China; 4 Department of General Surgery, Shanghai General Hospital, Shanghai Jiaotong University School of Medicine, Shanghai, China

## Abstract

The mechanisms underlying the role of CXCL5 in tumor angiogenesis have not been fully defined. Here, we examined the effect of CXCL5 on tumor angiogenesis in colorectal cancer (CRC). Immunohistochemistry was used to monitor the expression of CXCL5 and CD31 in CRC patients’ tissues. HUVEC cell lines stably transfected with shCXCR2 and shFOXD1 lentivirus plasmids were used in an in vitro study. Based on some molecular biological experiments in vitro and in vivo, we found that CXCL5 was upregulated in tumor tissues and that its level positively correlated with the expression of CD31. Next, we used recombinant human CXCL5 (rhCXCL5) to stimulate HUVECs and found that their tube formation ability, proliferation, and migration were enhanced by the activation of the AKT/NF-κB/FOXD1/VEGF-A pathway in a CXCR2-dependent manner. However, silencing of CXCR2 and FOXD1 or inhibition of the AKT and NF-κB pathways could attenuate the tube formation ability, proliferation, and migration of rhCXCL5-stimulated HUVECs in vitro. rhCXCL5 can promote angiogenesis in vivo in Matrigel plugs, and the overexpression of CXCL5 can also increase microvessel density in vivo in a subcutaneous xenotransplanted tumor model in nude mice. Taken together, our findings support CXCL5 as an angiogenic factor that can promote cell metastasis through tumor angiogenesis in CRC. Furthermore, we propose that FOXD1 is a novel regulator of VEGF-A. These observations open new avenues for therapeutic application of CXCL5 in tumor anti-angiogenesis.

## Introduction

Colorectal cancer (CRC) is the second most commonly diagnosed cancer in females and the third most commonly diagnosed cancer in males around the world^1^. Many breakthroughs have been made in the treatment of CRC over the past few decades, including postoperative adjuvant chemotherapy, perioperative chemotherapy, postoperative combined chemotherapy and radiotherapy, and targeted therapy. However, the mortality of CRC patients remains high. In 2016, there were 830,000 deaths from CRC^1^. Tumor metastasis, as the leading cause of death for most patients, is a multipathway and complicated process that requires the abilities of tumor migration and invasion, as well as tumor angiogenesis^2,3^. Because tumor angiogenesis plays a key role in tumor metastasis, and anti-angiogenesis therapy has become an important therapeutic strategy in CRC, it is of great importance to explore the mechanisms of angiogenesis in CRC.

CXCL5 is a member of the ELR+ CXC chemokine family, whose members contain a highly conserved three amino acid motif (ELR+) that promotes angiogenesis and is highly associated with aberrant angiogenesis^4,5^. Previous studies have reported that elevated levels of CXCL5 were detected in human non-small-cell lung cancer that was related to the vascularity of these tumors^4,6^. Antibody neutralization of CXCL5 in experimental models of human non-small-cell lung cancer decreased tumor angiogenesis and metastasis^7^. In addition, CXCL5 mediates several cellular functions, including neutrophil trafficking and tumor migration and invasion^8^. In our previous study, we demonstrated that CXCL5 is overexpressed and is associated with invasion, migration, and advanced tumor stages in CRC^2^. However, the mechanisms of its function in tumor angiogenesis in CRC are largely unknown.

In the present study, we found that the expression of CXCL5 was significantly correlated with CRC angiogenesis. Furthermore, we also examined the function of CXCL5 in angiogenesis in vitro and in vivo. In addition, we revealed that CXCL5 promoted angiogenesis via activating the AKT/NF-κB/FOXD1/vascular endothelial growth factor A (VEGF-A) pathway in a CXCR2-dependent manner. These observations suggest that CXCL5 may be a potential target for anti-angiogenesis therapy in CRC.

## Results

### CXCL5 overexpression in human CRC tissues is positively correlated with the microvessel marker CD31

**Fig. 1:**
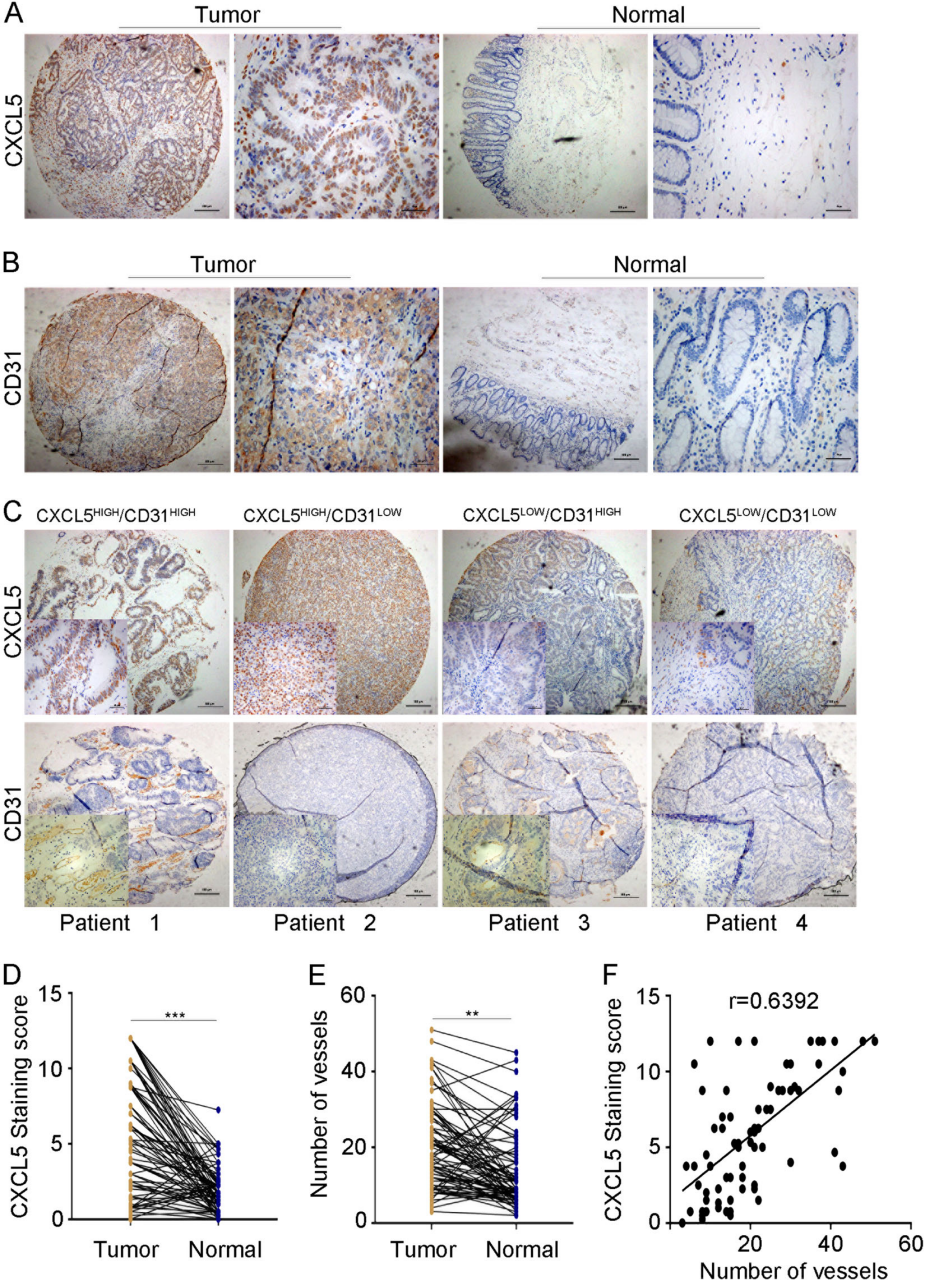
High expression of CXCL5 and CD31 in CRC tissues. **a**, **d** Immunohistochemistry images showing that CXCL5 is highly expressed in tissue microarray. **b**, **e** Immunohistochemistry images showing that CD31 is highly expressed in tissue microarray. **c**, **f** Correlation between CXCL5 and CD31 expression. CD31 expression is positively related with CXCL5 expression (*r* = 0.6392). Scale bars, 200 μm (magnification ×40) and 50 μm (magnification ×200). Data represent the mean ± SD, ^*^P < 0.05, ^**^P < 0.01, ^***^P < 0.001

Previously, we detected the expression of CXCL5 in CRC tissue microarrays, which included 78 pairs of CRC specimens, using immunohistochemical staining^2^. We selected a staining score of 4.5 as the cutoff value using the X-tile software as described in our previous article^2^. The expression of CXCL5 was upregulated in approximately 61.5% (48/78) in these paired tissue samples (Fig. 1a, d).

CD31, a transmembrane glycoprotein, is expressed by endothelial cells and many types of hematopoietic cells^9^. Previous reports have effectively documented that CD31 is a sensitive and specific endothelial marker in paraffin sections^10^. Thus, we examined the expression of CD31 in our CRC tissue microarray, and the results indicated that the expression of CD31 was increased in the tumor tissues compared with the normal tissues (*P* < 0.01, Fig. 1b, e).

We also analyzed the correlation between CXCL5 and CD31 expression. The results showed that high CXCL5 expression was positively correlated with CD31 expression (*r* = 0.6392, Fig. 1c, f). Together, these data indicate that CXCL5 may promote tumor angiogenesis in CRC.

### CXCL5 stimulates VEGF-A expression in HUVECs and promotes HUVEC angiogenesis through CXCR2

Because VEGF-A is generally regarded as a vital angiogenic factor^11^, we first investigated the secreted VEGF-A protein levels in human umbilical vein endothelial cell (HUVEC) culture media and the mRNA levels in HUVECs after treatment with recombinant human CXCL5 (rhCXCL5) over a concentration and time gradient using enzyme-linked immunosorbent assay (ELISA) and quantitative real-time polymerase chain reaction (qPCR). The VEGF-A secretion level increased and reached its maximum concentration after 36 h of stimulation at the optimal rhCXCL5 concentration of 10 ng/ml (Figure S1A, B). In agreement with the protein level, the mRNA levels showed the same results (Figure S1C, D).

**Fig. 2:**
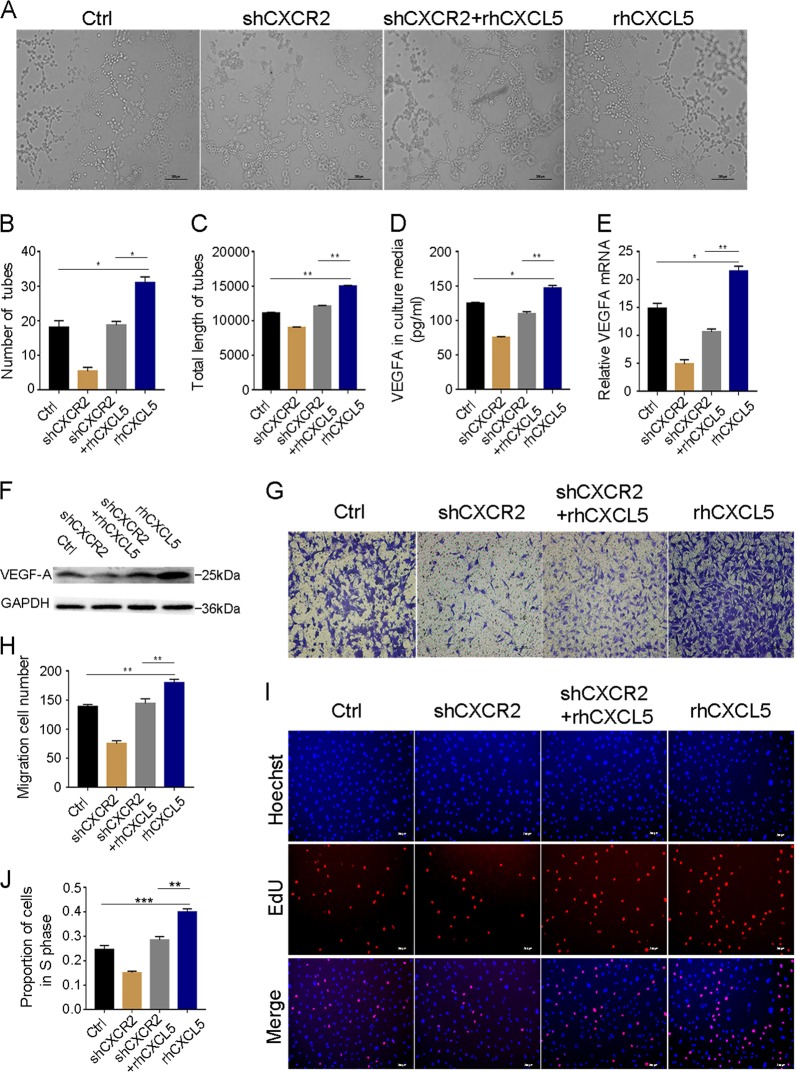
rhCXCL5 promotes HUVEC tube formation, migration, and proliferation through the CXCR2 pathway. **a** Images of HUVEC tube formation assay in different groups. Formation of tube-like networks was stimulated by the addition of rhCXCL5. Number of tubes and total length of tubes demonstrate the ability of angiogenesis in each group. Scale bars, 200 μm (magnification ×40). **b**, **c** Number of tubes and total length of tubes significantly increase in the rhCXCL5 group and decrease in the CXCR2-shRNA group. **d**–**f** VEGF-A protein and mRNA expression were detected by ELISA, western blot, and qPCR in different groups. rhCXCL5 increases VEGF-A expression which is inhibited by CXCR2-shRNA. **g** Images of transwell assay in different groups. Scale bar, 100 μm (magnification ×100). **h** Migration cell numbers are increased in the rhCXCL5 group compared with rhCXCL5-stimulated HUVEC-shCXCR2 group and control group. **i** EdU assay results in different groups. Scale bar, 200 μm (magnification ×40). **j** Proportion of cells in the S phase markedly increase in the rhCXCL5 group compared with rhCXCL5-stimulated HUVEC-shCXCR2 group and control group. Data represent the mean ± SD, ^*^P < 0.05, ^**^P < 0.01, ^***^P < 0.001

To further verify our hypothesis, we investigated whether CXCL5 could promote angiogenesis in vitro. We used rhCXCL5 to stimulate HUVECs and observed the effect on angiogenesis by performing tube formation assays. We found that the endothelial cell tube formation ability was increased after treatment with rhCXCL5 (Fig. 2a). Correspondingly, the number of tubes and the total length of the tubes were significantly increased in the rhCXCL5 group compared with the control group (*P* < 0.05 and *P* < 0.01, Fig. 2b, c).

CXCR2, the receptor of CXCL5, is responsible for ELR+ CXC chemokine-mediated angiogenic activity^12^. We next investigated whether the rhCXCL5-stimulated expression of VEGF-A in HUVECs was dependent upon CXCR2. To this end, CXCR2 was stably downregulated in HUVECs by CXCR2-shRNA, and the CXCR2 expression level was confirmed by western blot (Figure S1E). We found that the tube formation ability was decreased in rhCXCL5-stimulated HUVEC-shCXCR2 cells (Fig. 2a). The number of tubes and total length of the tubes were significantly decreased in the rhCXCL5-stimulated HUVEC-shCXCR2 group compared with the rhCXCL5 group (*P* < 0.05 and *P* < 0.01, Fig. 2b, c). Additionally, when HUVEC-shCXCR2 cells were incubated with rhCXCL5, low VEGF-A expression was detected in the supernatants using ELISA (*P* < 0.01), which was consistent with the results of the qPCR (*P* < 0.01) and western blot experiments (Fig. 2d–f).

Because the promoting effect on angiogenesis was usually acquired by promoting the migration and proliferation of vessel endothelial cells^13^, we next explored rhCXCL5’s influence on the migration and proliferation of HUVECs. As shown in Fig. 2g, h, the migration capacity of HUVECs was promoted by rhCXCL5 (*P* < 0.01), but the migration-promoting behavior was suppressed in rhCXCL5-stimulated HUVEC-shCXCR2 cells (*P* < 0.01). In addition, an EdU assay was used to evaluate the effect of rhCXCL5 on HUVEC proliferation. The results show that rhCXCL5 can significantly promote HUVEC proliferation (*P* < 0.001); however, this capability was inhibited in rhCXCL5-stimulated HUVEC-shCXCR2 cells (*P* < 0.001) (Fig. 2i, j). The results of the proliferation ability were also confirmed by a CCK8 assay (Figure S1F). In conclusion, these results indicate that CXCL5 can promote HUVEC angiogenesis through induction of VEGF-A through its receptor CXCR2.

### FOXD1 is involved in CXCL5/CXCR2-induced VEGF-A expression

**Fig. 3:**
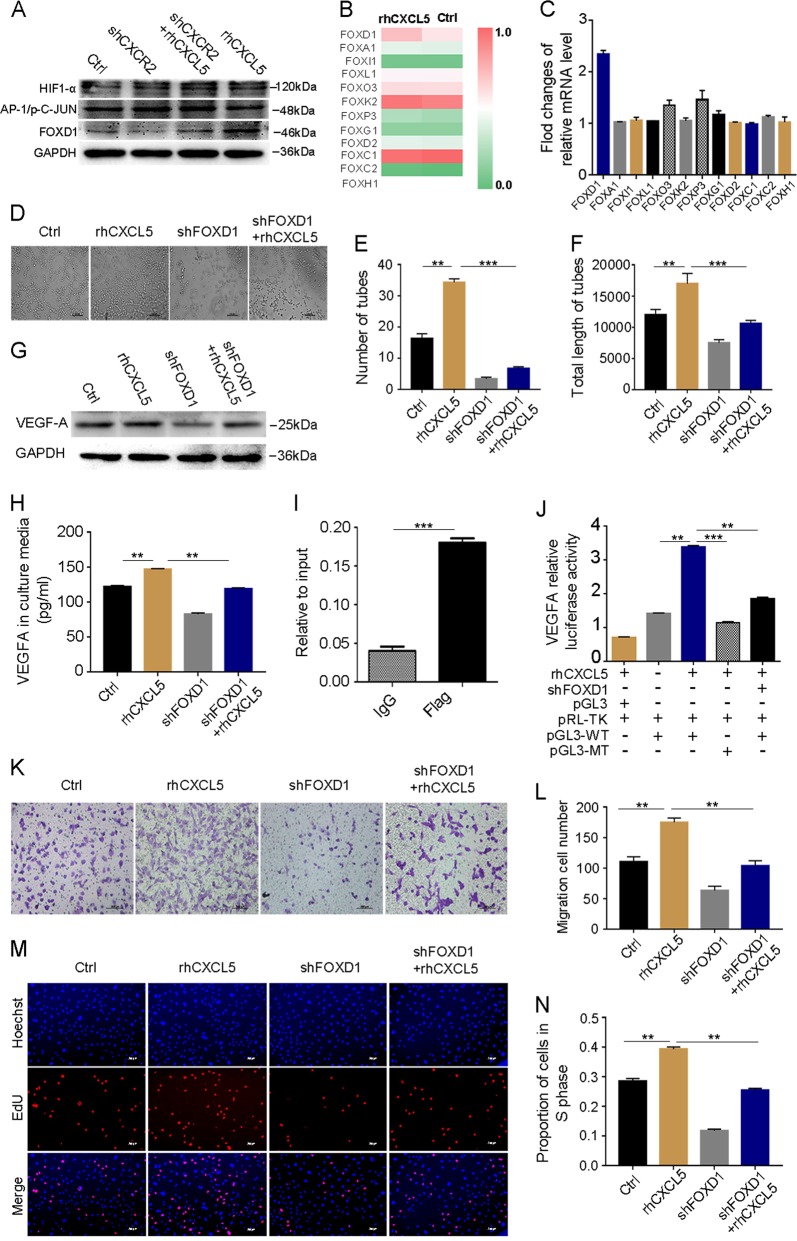
The CXCL5/CXCR2 induces the expression of VEGF-A dependent on FOXD1. **a** Western blot analyses of HIF-1α, C-JUN, and FOXD1 expression in the different groups. **b** A heat map displays some FOX protein expression level after being treated with or without rhCXCL5 in HUVECs. **c** Fold changes of the relative mRNA level of VEGF-A-related FOX gene after being treated with or without rhCXCL5 in HUVECs. **d** Images of HUVEC tube formation assay in each group. Scale bar, 200 μm (magnification ×40). **e**, **f** Number of tubes and total length of tubes in different groups, FOXD1 silencing in HUVECs significantly decreases HUVEC tube formation. **g**, **h** VEGF-A protein expression is examined by western blot and ELISA in different groups. **i** ChIP-qPCR assay using Flag antibody or control IgG in HUVECs transfected with a FOXD1 (Flag-tagged) plasmid shows the binding of FOXD1 on the VEGF-A promoter. **j** A reporter plasmid for VEGF-A (pGL3-VEGF-A) was generated by cloning the VEGF-A promoter region (WT) or its FOXD1 binding site mutants (MT) into the pGL3 basic vector. rhCXCL5 significantly increased the luciferase activity of the VEGF-A promoter region (WT), while the activity was significantly decreased when transfected with MT sequence. Meanwhile, VEGF-A luciferase activity was inhibited when HUVECs were transfected with the shFOXD1 plasmid. **k** Images of transwell assay in each group. Scale bars, 100 μm (magnification ×100). **l** Migration cell numbers were reduced by knocking down FOXD1 in HUVECs. **m** Images of the EdU assay in each group. Scale bars, 200 μm (magnification ×40). **n** Proportion of cells in the S phase were reduced by knocking down FOXD1 in HUVECs. Data represent the mean ± SD, ^*^P < 0.05, ^**^P < 0.01, ^***^P < 0.001

Previous studies have demonstrated that HIF-1α and AP-1 are the potential upstream transcription factors of VEGF-A^14,15^. To examine whether HIF-1α and (or) AP-1 can act as downstream effecting factors of rhCXCL5 to promote VEGF-A expression, we detected their expression levels using western blotting. As shown in Fig. 3a, the results validated that after HUVECs were treated with rhCXCL5, AP-1 and HIF-1α had little influence on VEGF-A secretion compared with the nontreated group. This result showed that CXCL5/CXCR2 did not regulate VEGF-A expression via HIF-1α and AP-1 transcription.

Some studies have reported that Forkhead box (FOX) proteins, a conserved family of transcription factors, regulate a wide range of biological processes, such as proliferation, migration, angiogenesis, differentiation, and apoptosis^16–18^, and that they participate in the occurrence and progression of tumors^19^. Thus, we hypothesized that one or some members of the FOX family may be the upstream transcriptional regulator of VEGF-A. To prove our hypothesis, we predicted some members of the FOX family from the JASPAR database and found that these members of the FOX family can bind to the VEGF-A promoter (Table S1). Next, qPCR was used to verify which one was mainly associated with the regulation of VEGF-A after HUVECs were treated with rhCXCL5. We found that FOXD1 was significantly upregulated after rhCXCL5 treatment (Fig. 3b, c). This result was also confirmed by western blot (Fig. 3a). To further investigate the function of FOXD1 in HUVEC angiogenesis and whether FOXD1 transcriptionally regulated the VEGF-A gene, we knocked down the expression of FOXD1 (Figure S2A). The increased ability of HUVECs to form tubes following rhCXCL5 treatment was suppressed after transfection with FOXD1-shRNA (Fig. 3d). Correspondingly, the number of tubes and total length of tubes were significantly decreased (*P* < 0.001, Fig. 3e, f). Moreover, as shown in Fig. 3g, h, western blot and ELISA assay validated that the VEGF-A protein levels were decreased in the rhCXCL5-stimulated HUVEC-shFOXD1 group compared with the rhCXCL5 group (*P* < 0.01).

Since there is a FOXD1 binding motif in the promoter region of the VEGF-A gene (Table S1), we next performed a ChIP-qPCR assay and verified that FOXD1 directly binds to the promoter region of the VEGF-A gene in HUVECs (Fig. 3i, Fig. S3A, B). Furthermore, the transcriptional regulation of VEGF-A by FOXD1 was also confirmed using a luciferase reporter assay, which demonstrated that the luciferase activity of VEGF-A was upregulated in rhCXCL5-stimulated HUVECs transfected with VEGF-A-WT (−1135 to −1128 nt) compared with the control cells without rhCXCL5 stimulation (*P* < 0.01). However, the activity was markedly decreased when the HUVECs were transfected with VEGF-A-MUT (*P* < 0.001). Moreover, the VEGF-A luciferase activity was also significantly reduced when HUVECs were transfected with FOXD1-shRNA (*P* < 0.01, Fig. 3j). Therefore, this result indicated that rhCXCL5 induced VEGF-A expression through the binding of FOXD1 protein to the VEGF-A promoter.

We also examined some biological functions, including migration and proliferation, in HUVEC-shFOXD1 cells. As shown in Fig. 3k, l, the migration-promoting behavior of rhCXCL5 was suppressed when HUVECs were transfected with FOXD1-shRNA (*P* < 0.01). In addition, an EdU assay was used to assess the effect of rhCXCL5 on HUVEC-shFOXD1 cell proliferation, and we found that the proliferative ability of HUVECs was suppressed in the rhCXCL5-stimulated HUVEC-shFOXD1 group compared with the rhCXCL5 control group (*P* < 0.01, Fig. 3m, n). The proliferative ability was also confirmed using a CCK8 assay (Figure S2B). In conclusion, these results indicate that CXCL5/CXCR2 promotes HUVEC angiogenesis through binding of FOXD1 protein to the VEGF-A promoter.

### FOXD1 activity is regulated by the CXCL5/CXCR2 axis via the AKT/NF-κB pathway

**Fig. 4:**
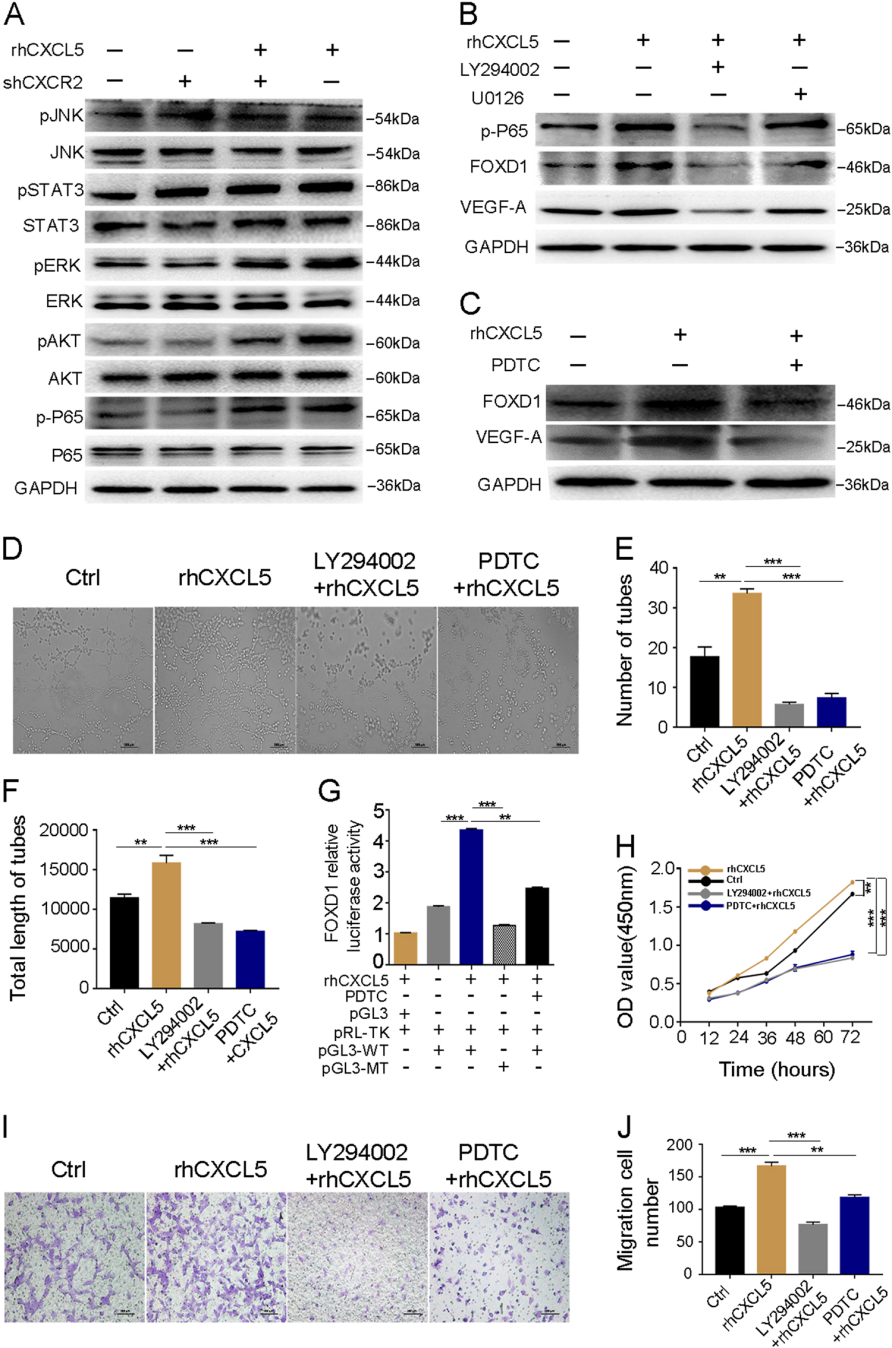
CXCL5/CXCR2 axis promotes FOXD1 activity through the AKT/NF-κB pathway. **a** Screening of CXCL5/CXCR2 downstream signaling pathway using western blot after different treatments in HUVECs. **b** Western blot shows that the AKT pathway rather than the ERK pathway regulates the expression of FOXD1 and VEGF-A using pathway inhibitors U0126 and LY294002. **c** Western blot analyses of p-P65, FOXD1, and VEGF-A in indicated groups after using the NF-κB inhibitor PDTC. **d** Images of HUVEC tube formation in indicated groups. Scale bars, 200 μm (magnification ×40). **e**, **f** Number of tubes and total length of tubes in different groups. Inhibitor of LY294002 and PDTC obviously inhibited the ability of HUVEC tube formation. **g** A reporter plasmid for FOXD1 (pGL3-FOXD1) was generated by cloning the FOXD1 promoter region (WT) or its NF-κB binding site mutants (MT) into the pGL3 basic vector. rhCXCL5 significantly increased the luciferase activity of the FOXD1 promoter region (WT), while the activity was significantly decreased when transfected with MT sequence. Meanwhile, FOXD1 luciferase activity can be inhibited by PDTC. **h** CCK8 assay in different groups. Inhibitor of LY294002 and PDTC noticeably reduced HUVEC proliferation ability. **i** Images of transwell assay in different groups. Scale bars, 100 μm (magnification ×100). **j** The migration ability of HUVECs was suppressed by the inhibitor of LY294002 and PDTC. Data represent the mean ± SD, ^*^P < 0.05, ^**^P < 0.01, ^***^P < 0.001

It has been reported that the AKT, ERK, JNK, and STAT3 pathways were related to the evolution and progression of carcinoma mediated by the CXCL5/CXCR2 axis^20,21^. In an attempt to explore how FOXD1 is regulated by CXCL5/CXCR2, we examined the activity of these pathways. As shown in Fig. 4a, rhCXCL5 activated the ERK and AKT pathways, as indicated by the upregulation of pERK and pAKT. In addition, the knockdown of CXCR2 suppressed the phosphorylation of ERK and AKT, as shown by decreased levels of pERK and pAKT. To verify which of these two pathways is related to the CXCL5/CXCR2 axis-induced upregulation of FOXD1 expression, we used inhibitors of the ERK (U0126) and AKT (LY292004) pathways to block their activity. Our results indicated that inhibition of the AKT pathway, rather than the ERK pathway, changed the expression of FOXD1 and VEGF-A (Fig. 4b). A previous study reported that NF-κB was a downstream effector of the AKT pathway^22^. We next examined the alteration of the NF-κB pathway. Western blotting validated that rhCXCL5 activated the NF-κB pathway as shown by the upregulation of p-P65 (Fig. 4a). However, the protein level of p-P65 was significantly decreased in the HUVEC-shCXCR2 group and HUVECs after treatment with LY294002 (Fig. 4a, b). Moreover, we treated HUVECs with PDTC, an inhibitor of NF-κB and found that PDTC suppressed the rhCXCL5-dependent induction of FOXD1 and VEGF-A expression (Fig. 4c). Based on these findings, the effects of these two pathways on HUVEC angiogenesis were evaluated by tube formation assays. The result revealed that the AKT (LY294002) and NF-κB (PDTC) inhibitors clearly block the tube formation ability of HUVECs (Fig. 4d). Correspondingly, the number of tubes and total length of the tubes were significantly decreased (*P* < 0.001, Fig. 4e, f).

To further determine whether NF-κB is directly involved in rhCXCL5-induced FOXD1 protein expression, we performed a luciferase assay to measure the FOXD1 promoter luciferase activity upon expression of NF-κB. As expected, rhCXCL5 promoted FOXD1 expression in HUVECs transfected with FOXD1-WT (−960 to −951 nt) (*P* < 0.001). However, the FOXD1 luciferase activity was markedly decreased in HUVECs transfected with FOXD1-MUT (*P* < 0.001). Moreover, the FOXD1 luciferase activity was also significantly reduced when HUVECs were treated with PDTC (*P* < 0.01, Fig. 4g).

According to the results of the analyses of the signaling pathways, some biological behaviors, such as proliferation and migration, were also examined. A CCK8 assay was used to evaluate the proliferative ability of HUVECs. The results indicated that the AKT (LY294002) and NF-κB (PDTC) inhibitors noticeably reduced HUVEC proliferation (*P* < 0.001, Fig. 4h). In addition, the migration ability of HUVECs was also suppressed by the LY294002 (*P* < 0.001) and PDTC (*P* < 0.001) inhibitors (Fig. 4i, j). Taken together, these results suggested that the CXCL5/CXCR2-regulated FOXD1 activity is mediated by the AKT/NF-κB pathway.

### The CXCL5/CXCR2 axis promotes tumor angiogenesis in vivo

**Fig. 5:**
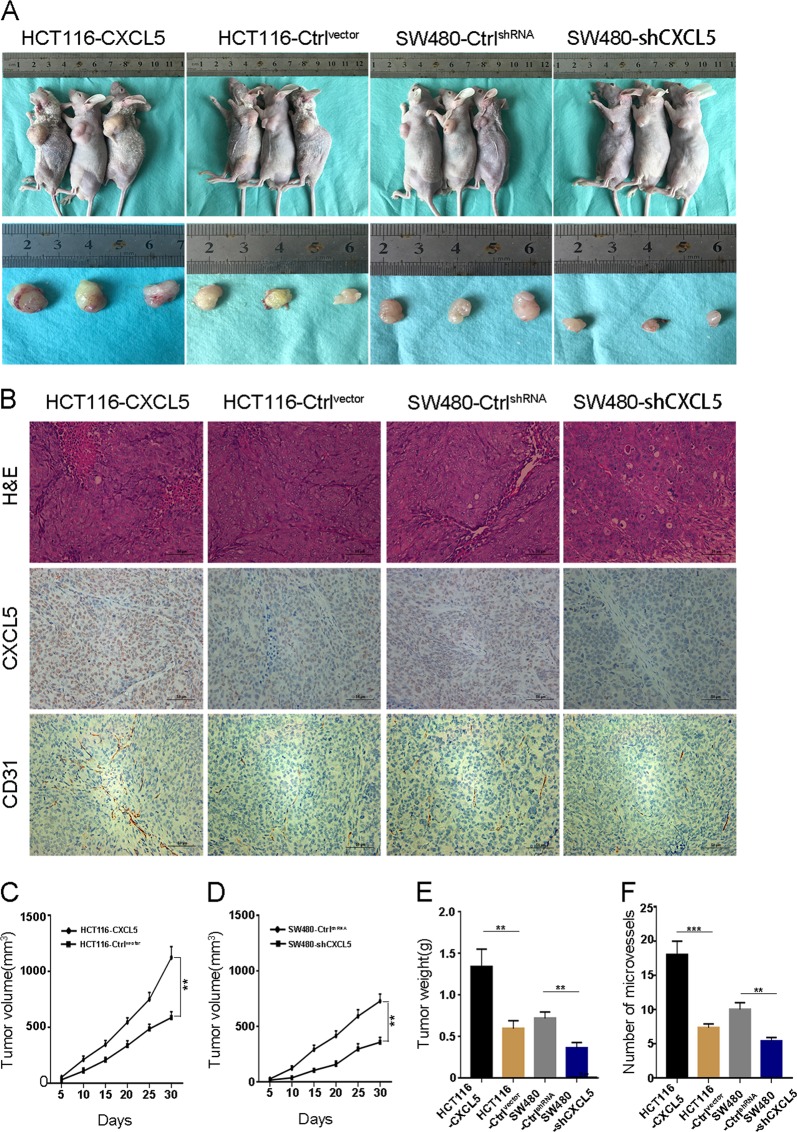
CXCL5 promotes subcutaneous tumor growth and angiogenesis in nude mice. (**a**) Representative images of tumor-bearing mice and tumor mass. (**b**) Representative images of H&E staining as well as IHC staining of CXCL5 and CD31 of subcutaneous tumor in nude mice. Scale bars, 50 μm (magnification 200×). (**c**, **d**) Tumor growth curves. The volumes of xenografts were measured every 5 days during a 30-day period. (**e**) Average tumor weight of each group. (**f**) Number of microvessels in each group. Data represent the mean ± SD, *P < 0.05, **P < 0.01, ***P < 0.001

To verify whether CXCL5 overexpression or knockdown could regulate CRC growth and tumor angiogenesis in vivo, we used the HCT116-CXCL5, HCT116-Control^vector^, SW480-shCXCL5, and SW480-Control^shRNA^ cells that were constructed in our previous studies to generate a subcutaneous xenotransplanted tumor model in nude mice. As shown in Fig. 5a, c, d, the growth rates of the HCT116-CXCL5 and SW480-Control shRNA cells were significantly faster compared with the HCT116-Control vector and SW480-shCXCL5 cells. In addition, the average weights of the tumors generated from HCT116-CXCL5 and SW480-Control^shRNA^ cells were significantly increased in comparison with the tumors from the HCT116-Control^vector^ and SW480-shCXCL5 cells (1.337 ± 0.123 vs. 0.592 ± 0.056, *P* < 0.01 and 0.716 ± 0.045 vs. 0.357 ± 0.040, *P* < 0.01; Fig. 5e).

Furthermore, to determine the role of CXCL5 in tumor angiogenesis, the tumor nodules were stained for CXCL5 and the microvessel marker CD31. The immunohistochemistry (IHC) results revealed that CD31-marked vessels were more abundant in HCT116-CXCL5 and SW480-Control^shRNA^ groups than in the HCT116-Control^vector^ and SW480-shCXCL5 groups (*P* < 0.001 and *P* < 0.01, Fig. 5b, f).

### CXCL5 enhanced angiogenesis in Matrigel plugs in vivo

**Fig. 6:**
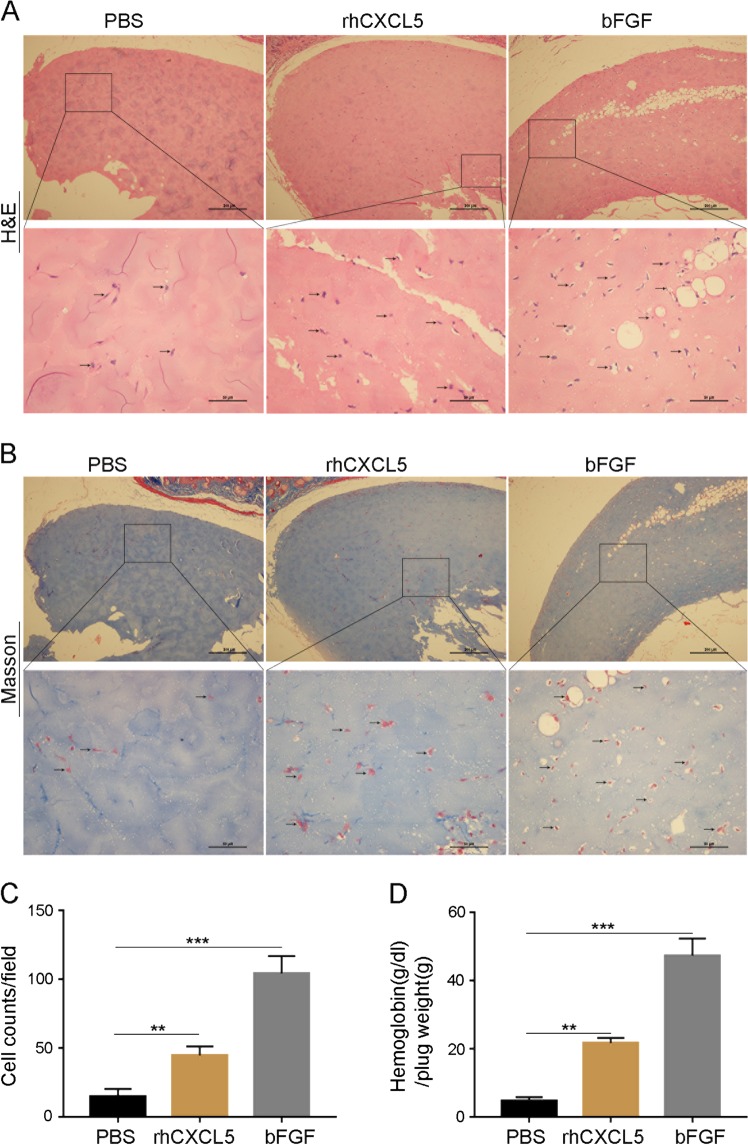
CXCL5 enhances blood vessel growth in Matrigel plugs in vivo. **a** Representative photographs of the Matrigel plug sections stained with H&E. Number of cells manifest the ability of angiogenesis in different groups. Arrows on the lower columns indicate areas of blood cells. Scale bars, 50 μm (magnification ×200). **b** Representative photographs of the Matrigel plug sections stained with Masson’s trichrome. Number of cells manifest the ability of angiogenesis in different groups. Arrows on the lower columns indicate areas of blood cells. Scale bars, 50 μm (magnification ×200). **c** Cells migrating to form microvessels in Matrigel containing rhCXLC5 (10 ng) were more than PBS control. **d** Hemoglobin content of the rhCXCL5- (10 ng) treated group was significantly great compared with that of the PBS control. Matrigel containing bFGF (100 ng) served as a positive control. Data represent the mean ± SD, ^*^P < 0.05, ^**^P < 0.01, ^***^P < 0.001

**Fig. 7:**
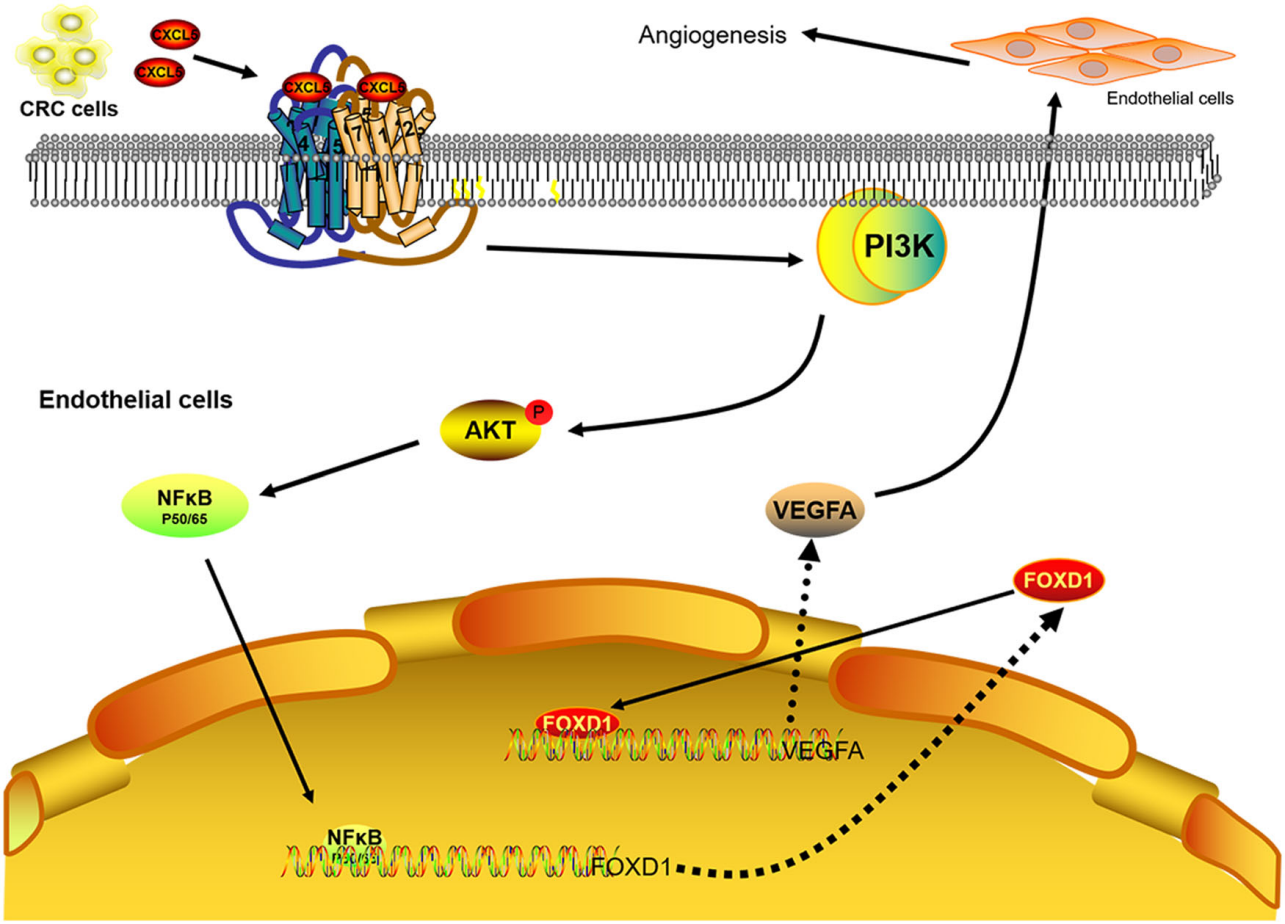
Proposed model for the regulation of tumor angiogenesis by CXCL5. CXCL5 increases AKT phosphorylation in a CXCR2-dependent manner and then NF-κB translocates to the nucleus. This leads to FOXD1 transcriptional activity that is enhanced at the VEGF-A promoter. Consequently, the expression of VEGF-A is increased to promote tumor angiogenesis

The influence of CXCL5 on tumor angiogenesis in vivo was also evaluated using Matrigel plugs. The plugs were examined histologically employing H&E (Fig. 6a) and Masson’s trichrome staining (Fig. 6b). The Matrigel sections with rhCXCL5 contained many more cells than similar Matrigel sections with PBS and contained cells with a similar morphology to those in the bFGF (100 ng) control (*P* < 0.01, Fig. 6c). Furthermore, the hemoglobin levels were also measured, and the results indicated that the hemoglobin content of the rhCXCL5-treated group was higher than that in the PBS control (*P* < 0.01, Fig. 6d). These results supported the role of CXCL5 in angiogenesis in vivo. A schematic diagram of our study is shown in Fig. 7.

## Discussion

The major cause of death in cancer patients is from metastasis. There is ample evidence that several chemokines and their receptors play important roles in the metastatic process^8^. In our previous study, we demonstrated that CXCL5 was upregulated in CRC and was able to promote tumor metastasis through induction of EMT in CRC tumor cells^2^. Angiogenesis is an important step in the progression and maintenance of a malignant phenotype. Previous work has verified that CXCL5 is a vital angiogenic factor in non-small-cell lung cancer^7^. However, very little information is available regarding the effects of CXCL5 on tumor angiogenesis in CRC. Thus, in the present study, we confirmed that CXCL5 promotes tumor angiogenesis both in vitro and in vivo. We found that CXCL5 staining was markedly stronger in tumor tissues and that it was positively associated with CD31 staining. Tumor angiogenesis is a sophisticated process involving the degradation of the basement membrane, and the proliferation and migration of endothelial cells^23^. Our findings validated that in vitro, CXCL5 enhanced proliferation, migration, and tube formation of HUVECs. We next knocked down CXCR2, the receptor of CXCL5, in HUVEC cell lines. The proliferation, migration, and tube formation were impaired in the CXCR2-knockdown group, suggesting that CXCL5 promoted angiogenesis in a CXCR2-dependent manner. In addition, CXCL5 also enhanced tumor angiogenesis in vivo, which was confirmed by a subcutaneous xenotransplanted tumor model and Matrigel plugs in nude mice.

Another critical molecule in tumor angiogenesis is VEGF-A. Many studies have documented that VEGF-A is an important regulator of angiogenesis and mediates most of the steps in the angiogenic cascade in endothelial cells, including proliferation, migration, and tube formation^11^. In this study, we found a significant increase of VEGF-A secretion into the culture medium of HUVECs after treatment with rhCXCL5, and this phenotype also validated by western blot. Interestingly, the level of VEGF-A expression was suppressed when CXCR2 expression was knocked down in HUVECs. Therefore, we concluded that the CXCL5/CXCR2 axis must have an indirect relationship with VEGF-A expression.

Some studies have shown that VEGF-A expression is mediated by HIF-1α and AP-1^14,15^. However, in our experiment, we found that CXCL5/CXCR2-induced VEGF-A expression had no significant association with HIF-1α or AP-1. In reference to previous research, there are some FOX proteins, including, for example, FOXO, FOXC, and FOXP, which have been linked to tumorigenesis and tumor angiogenesis in certain cancers^16,19^. Therefore, we predicted possible relationships between several FOX protein subfamilies and VEGF-A using the JASPAR database, and confirmed that FOXD1 was strikingly associated with the CXCL5/CXCR2 axis in controlling VEGF-A secretion. Moreover, we found that the expression of VEGF-A was markedly decreased when FOXD1 expression was knocked down. This finding was in consistent with the HUVEC tube formation assay in the FOXD1-knockdown group in vitro. Our data show that CXCL5 promotes angiogenesis by increasing FOXD1 expression and transcription of its downstream target, VEGF-A. In summary, we suggest that FOXD1 is a novel upstream regulator of VEGF-A in angiogenesis.

To further investigate the mechanisms of the CXCL5/CXCR2 axis and FOXD1/VEGF-A, we screened some downstream signaling pathways of the CXCL5/CXCR2 axis, including the AKT, ERK, JNK, and STAT3 pathways^20,21^. In this study, we discovered that both the AKT and ERK pathways can be activated by rhCXCL5. Next, inhibitors of both pathways were used to determine which pathway is most important. The results showed that the AKT pathway, rather than the ERK pathway, had a major effect on the CXCL5/CXCR2 axis-induced FOXD1 and VEGF-A expression. It has been previously shown that AKT signaling can activate several downstream signaling molecules through phosphorylation^24^. NF-κB is one of these factors^22^. In our study, we confirmed that rhCXCL5 can promote NF-κB phosphorylation, but that the protein level of p-P65 was inhibited by an inhibitor of the AKT pathway. In addition, an NF-κB inhibitor significantly decreased FOXD1 and VEGF-A expression. Based on the above results, our data show that CXCL5/CXCR2 mediates VEGF-A expression through AKT phosphorylation and further promotes NF-κB phosphorylation and translocation to activate the FOXD1 gene.

In our study, we revealed that FOXD1 is a novel regulator of VEGF-A expression. Related studies show that FOXD1 is associated with organization development^25,26^, cell reprogramming^27^, and cell differentiation^28^, as well as tumorigenesis and progression^29,30^. However, the function of FOXD1 in tumor angiogenesis is poorly understood. In the present study, we confirmed that FOXD1 is a transcription factor for VEGF-A, which can be mediated by the CXCL5/CXCR2 axis. It has been reported that CXCL5 expression has been used as a prognosis predictor that is related to poor prognosis in many cancers, such as prostate cancer, endometrial cancer, hepatocellular carcinoma, and pancreatic cancer^31,32^. Previously, we have demonstrated that high CXCL5 expression is associated with poor prognosis in CRC^2^. Furthermore, our results also confirmed that CXCL5 overexpression enhanced tumor volume and tumor angiogenesis in an in vivo model. Actually, human VEGF-A has been shown to effectively induce angiogenesis in many other species due to their structural homology, including rodents, rabbits, and pigs^33^. Specifically, many researches have demonstrated that human VEGF-A can promote tumor angiogenesis in mouse^34,35^; vice versa, the inhibition of human VEGF-A will suppress the tumor angiogenesis in mouse^36^. Therefore, it is reasonable to investigate human VEGF-A related questions in angiogenesis in mouse models. Taken together, our data could be a strong evidence that CXCL5 has a tumor-promoting effect in CRC as a positive regulator of tumor angiogenesis both in vitro and in vivo.

In conclusion, our study revealed that CXCL5 promotes tumor angiogenesis by controlling FOXD1 expression and transcriptional activity through the activation of the AKT/NF-κB pathway, suggesting that CXCL5 is a significant predictor in tumorigenesis and progression. Thus, the inhibition of the CXCL5/CXCR2 axis may be a promising treatment for CRC patients.

## Materials and methods

### Cell lines

The HUVEC cell lines were gifted from the Shanghai Institute of Intensive Care Medicine. HCT116-CXCL5, HCT116-Control^vector^, SW480-Control^shRNA^, and SW480-shCXCL5 cells were constructed in our previous studies^2^. Cells were cultured at 37 °C with 5% CO_2_ in DMEM medium containing 10% fetal bovine serum with 10^7^ U/L penicillin and 10 mg/L streptomycin in an incubator.

### Lentivirus vectors and endothelial cell transfection

The lentiviral vector LV3-pGLV-h1-GFP/puro-shCXCR2, LV3-pGLV-h1-GFP/puro-shFOXD1, and LV5-EF1a-GFP/Puro-FOXD1(FLAG-tag) were purchased from the Shanghai GenePharma Corporation (Shanghai, China). Cells in an exponential growth phase were subcultured in six-well plates to ensure 50% cell confluence by the next day for transfection. Lentivirus transfection was performed as described previously^3^. The targeting sequences are listed in Supplementary Table 2.

### Tissue microarray and IHC

Tissue microarray was manufactured by Shanghai Outdo Biotechnology Corporation, as described in our previous study^2^, and IHC staining was performed as previously described^3^. Briefly, the percentage of immunoreactive cells scoring was documented as 0 (none), 1 (<10%), 2 (10–50%), 3 (51–80%), and 4 (>80%). The intensity of immunostaining was scored as 0 (no immunostaining), 1 (weak immunostaining), 2 (moderate immunostaining), and 3 (strong immunostaining). The percentage of immunoreactive cells score and the intensity of the staining score were multiplied to generate the immunoreactivity score, which was evaluated by two pathologists independently.

### Enzyme-linked immunosorbent assay

ELISA (Cat# ELH-VEGF-1, Ray-Biotech, Norcross, GA, USA) analyses of VEGF-A were performed in gradient concentration and time post stimulation with recombinant human CXCL5 (rhCXCL5). The culture supernatants were collected, and VEGF-A secretion was quantified through ELISA according to the manufacturer’s protocol. Absorbance at 450 nm was detected using an Epoch Microplate Spectrophotometer (Bio Tek) after adding 50 μl Stop Solution.

### Quantitative real-time PCR

Total RNA was isolated from HUVECs using TRIzol reagent (Invitrogen, Carlsbad, CA, USA) according to the manufacturer’s instructions. cDNA was synthesized using a reverse transcription kit (Invitrogen, Carlsbad, CA, USA). Primers are listed in Supplementary Table 3. Then, PCR reactions were performed using an Applied Biosystems 7500 System and the SYBR Green Reagent kit (Applied Biosystems, Foster City, CA, USA), and GAPDH was used as a constitutive control. PCRs for each sample were conducted in triplicate.

### Western blot analysis

Cells were harvested and lysed by RIPA buffer (Solarbio, Beijing, China) containing 1% PMSF protease inhibitors and phosphatase inhibitors. Equal amounts of protein (60 μg) were electrophoresed on 12.5% sodium dodecyl sulfate polyacrylamide gel electrophoresis gel and electrotransferred to polyvinylidene fluoride membranes. The membranes were blocked with 5% bovine serum albumin for 2 h and then were incubated with primary antibodies at 4 °C overnight. The primary antibodies for CXCR2, VEGF-A, FOXD1, and HIF-1α were purchased from Abcam, and anti-FLAG, anti-JNK, anti-p-JNK, anti-ERK, anti-p-ERK anti-AKT, anti-p-AKT, anti-STAT3, anti-p-STAT3, anti-P65, anti-p-P65, as well as anti-AP-1 were purchased from Cell Signaling Technology. Then, the membranes were washed three times and incubated with secondary antibodies for 1 h at room temperature. Band intensities were quantitated by an enhanced chemiluminescence detection system (Amersham Bioscience, Piscataway, NJ, USA) according to the manufacturer’s protocol.

### Chromatin immunoprecipitation PCR (ChIP-qPCR) assay

To evaluate the interaction between FOXD1 and the VEGF-A promoter region, ChIP-qPCR assays were performed using a kit (Millipore) according to the manufacturer’s instructions. HUVECs transfected with a FOXD1 (FLAG-tag) plasmid were fixed with formaldehyde for protein/DNA cross-linking and then lysed. DNA fragmentation was performed by sonication on ice (25 pulses, 5 s on, 10 s off, 13 min) and then added to a well coated with an anti-FLAG antibody or control IgG at 4 °C overnight. The FOXD1 (FLAG-tag) bound DNA was released through protein digestion with proteinase K. The DNA was purified by passing a column. PCR reactions were performed using three pairs of different primers designed to target the VEGF-A gene. The primers are listed in Supplementary Table 4.

### Luciferase reporter assay

The human VEGF-A or FOXD1 gene promoters were cloned into the multi-cloning site of a pGL3 reporter plasmid containing firefly luciferase reporter gene (pGL3-WT-VEGF-A/FOXD1). Mutations were introduced into the sequences of the VEGF-A or FOXD1 promoter to generate VEGF-A or FOXD1 mutation reporters (pGL3-MT-VEGF-A/FOXD1). pRL-TK contains Renilla luciferase. Cells were plated into 24-well plates, and then co-transfected with different plasmids in serum-free medium. After 5 h of incubation, the supernatants were replaced with fresh complete medium with rhCXCL5 (0 or 10 ng/ml) or (and) PDTC. The cells were lysed 48 h later, mixed with the dual luciferase assay reagent (Promega, USA). Relative luciferase activity was calculated by normalizing the firefly luminescence to the Renilla luminescence.

### Endothelial tube formation assay

HUVECs transfecting with different plasmids or treating by the inhibitors of AKT (LY294002) and NF-κB (PDTC) were cultured in DMEM medium with or without rhCXCL5 (10 ng/ml). After 36-h incubation at 37 °C with 5% CO_2_, cells were plated in 96-well plates coated with 50 μl of Matrigel (BD company, USA) at a concentration of 4 × 10^4^ cells per well. Tubules were photographed through microscopy and computed by Image Pro Plus software after incubating for 4 h at 37 °C with 5% CO_2_.

### BeyoClick^TM^ EdU-555 cell proliferation assay

EdU assay was conducted using BeyoClick^TM^ EdU-555 cell proliferation Kit with Alexa Fluor 555 (Beyotime Biotechnology, Shanghai, China). HUVECs transfecting with different plasmids were cultured in DMEM medium with or without rhCXCL5 (10 ng/ml) in 24-well plates at an appropriate density at 37 °C, 5% CO_2_ for 36 h. Then, the assay was completed according to the manufacturer’s protocol. The cells were counted under a fluorescence microscope.

### CCK8 assay

Cell counting Kit-8 (CCK8) was used to evaluate cell proliferation. HUVECs transfecting with different plasmids or treating by inhibitors of AKT (LY294002) and NF-κB (PDTC) were plated in 96-well plates at a concentration of 2000 cells per well. rhCXCL5 (0 or 10 ng/ml) was used to stimulate the endothelial cells. Then, cell proliferation was measured every 12 h at an absorbance of 450 nm using Epoch Microplate Spectrophotometer (Bio Tek).

### Cell migration assay

HUVECs were transfected with different plasmids or treated by inhibitors of AKT (LY294002) and NF-κB (PDTC). In total, 1 × 10^5^ cells were added to serum-free DMEM medium with or without 10 ng/ml rhCXCL5 and plated in transwell chambers (8 μm for a 24-well plate; Corning Costar, NY, USA). Cells were fixed with 10% methanol and stained by 0.5% crystal violet after 16-h incubation. Then, cells were counted under an inverted microscope in the lower chamber.

### Mouse xenograft tumor models

CRC cells suspension (1 × 10^6^) were subcutaneously injected into 4-week-old male BALB/c nude mice (Institute of Zoology, China Academy of Sciences) as previously described^3^. Tumor nodules were measured every 5 days. Mice were killed and tumor nodules were collected for analysis.

### Matrigel plug assay in vivo

Matrigel plug assay has been performed as previously described^37,38^. Four-week-old BALB/c nude mice were injected subcutaneously with 500 μl of Matrigel containing PBS or bFGF (100 ng) as negative or positive controls and rhCXCL5 (10 ng) served as the experimental condition. After 10 days, mice were killed; Matrigel plugs were dissected out and analyzed for vascularity by hemoglobin measurement or by histology. For hemoglobin measurement, plugs were weighed and homogenized, and a serial dilution of methemoglobin was prepared for quantification purposes^39,40^. Fifty microliters of supernatants or standards were added to a 96-well plate in duplicate and 50 μl of tetramethylbenzidine was added to each sample. The plate was allowed to develop at room temperature, and absorbance was read with an ELISA plate reader at 450 nm. To calculate hemoglobin concentrations, the values (g/dl) were normalized to the weights of the plugs (g)^34,35^. Histology sections were manufactured by Shanghai Outdo Biotechnology Corporation. Matrigel sections from different treatment groups were examined by H&E or Masson’s trichrome staining and scored by two blinded observers (Zong Y.P. and Ou B.C.) to determine whether the Matrigel sections with rhCXCL5 contain more cells.

### Statistical analysis

Data are represented as mean ± SD. Statistical analyses were performed using SPSS 16.0 software (SPSS Inc.). The differences between the two groups were analyzed by the Student’s *t* test. All experiments were performed in triplicate. *P* < 0.05 was considered as statistically significant.

## Supplementary information

Figure S1

Figure S2

Figure S3

supplementary table

Supplemental figure legends
