# Supplementary figures and images for: CXCL5 induces tumor angiogenesis via enhancing the expression of FOXD1 mediated by the AKT/NF-κB pathway in colorectal cancer
Source: Cell Death Dis. 2019 Feb 21;10(3):178. doi: 10.1038/s41419-019-1431-6 (PMC6385313; doi:10.1038/s41419-019-1431-6)

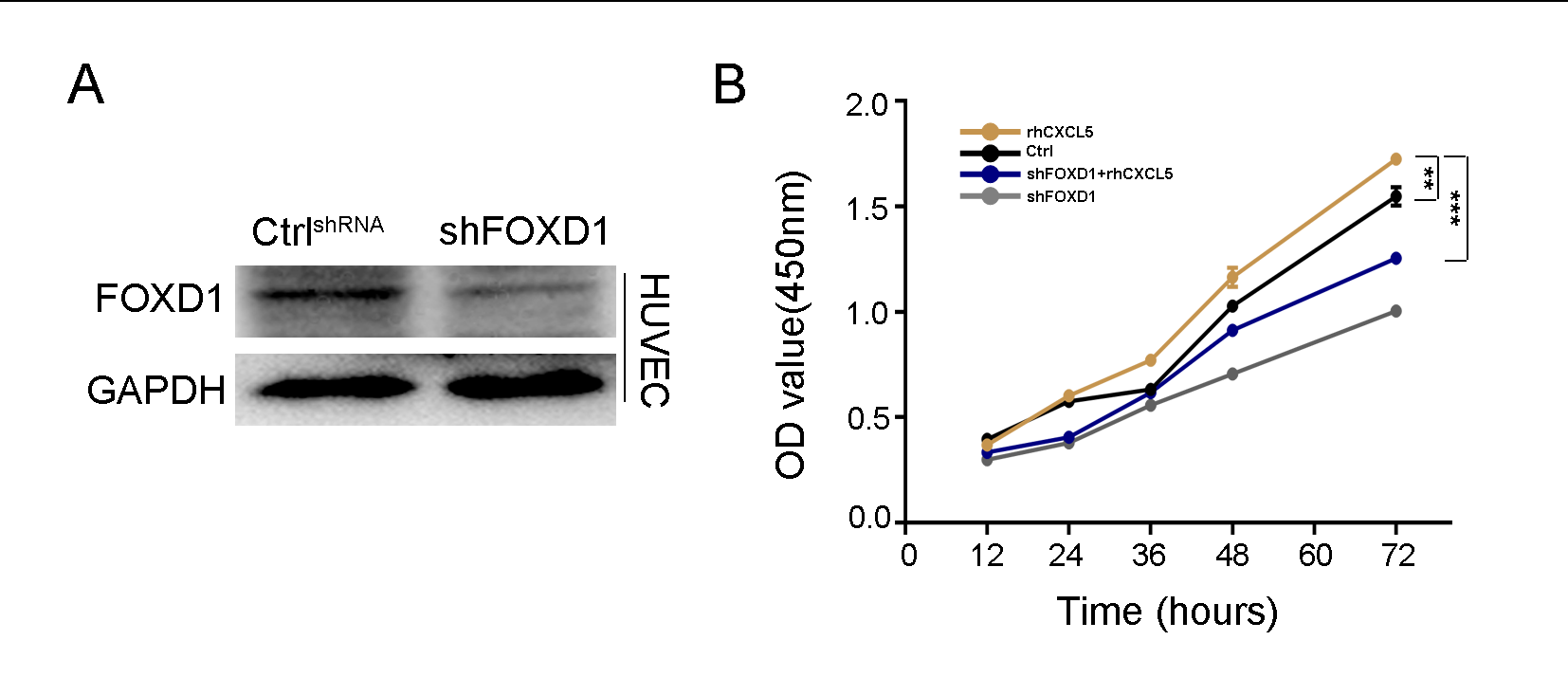

Supplement: Supplementary file 2 — Figure S2 [file 41419_2019_1431_MOESM2_ESM.tif]

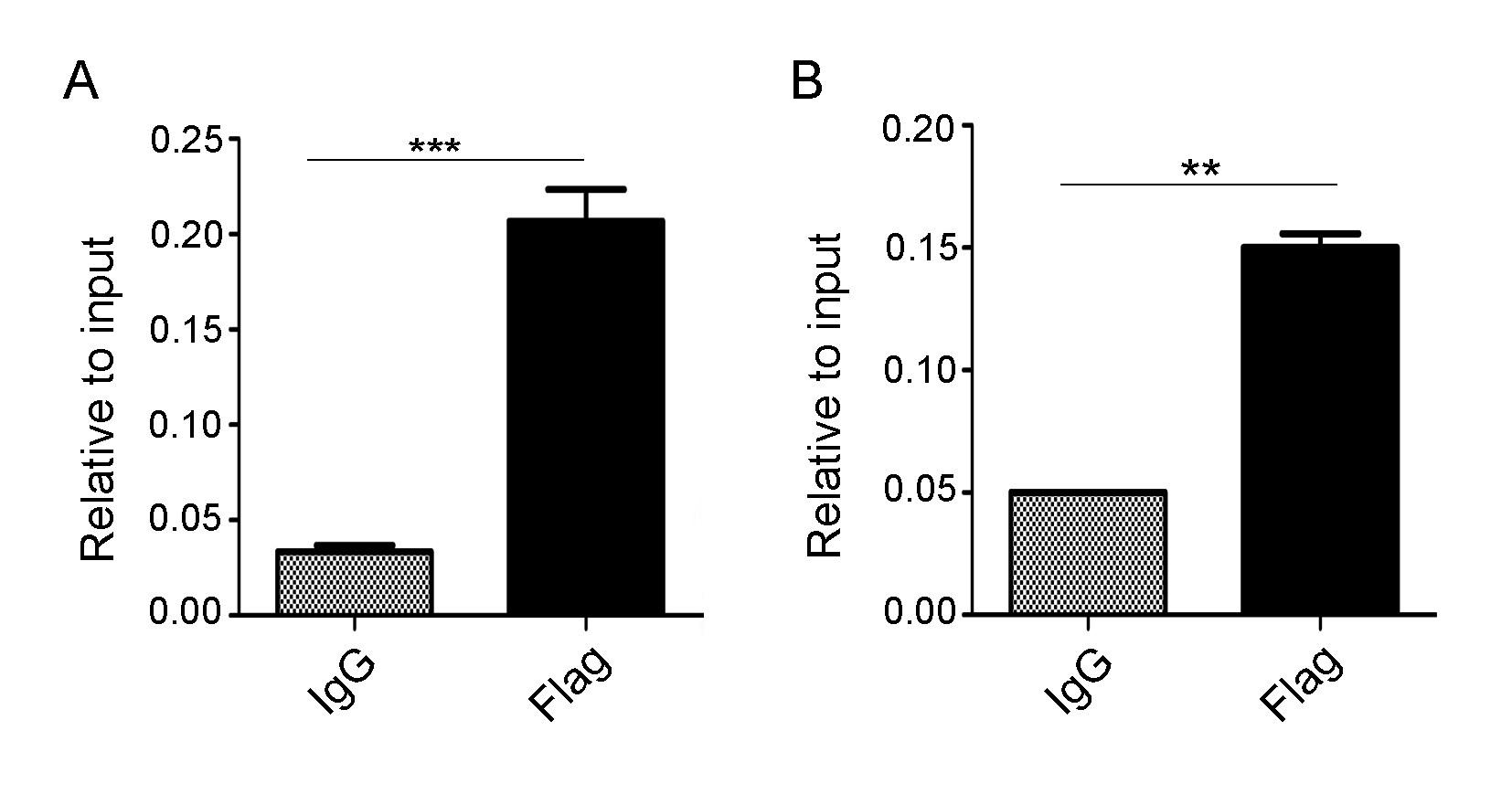

Supplement: Supplementary file 3 — Figure S3 [file 41419_2019_1431_MOESM3_ESM.tif]
