# Supplementary material for: CXCL5 induces tumor angiogenesis via enhancing the expression of FOXD1 mediated by the AKT/NF-κB pathway in colorectal cancer
Source: Cell Death Dis. 2019 Feb 21;10(3):178. doi: 10.1038/s41419-019-1431-6 (PMC6385313; doi:10.1038/s41419-019-1431-6)
Supplement: Supplementary file 4 — supplementary table [file 41419_2019_1431_MOESM4_ESM.docx]

**Table S1. FOX family binding sites to VEGF-A gene.**

| Matrix ID | Name | Score | Relative score | Sequence ID | Start | End | Strand | Predicted sequence |
| --- | --- | --- | --- | --- | --- | --- | --- | --- |
| MA0148.2 | FOXA1 | 11.4406 | 0.915452 | VEGFA | 885 | 895 | + | tgttggctctt |
| MA0032.2 | FOXC1 | 9.21996 | 0.891892 | VEGFA | 1125 | 1135 | + | gaaataaacat |
| MA0846.1 | FOXC2 | 10.2884 | 0.893945 | VEGFA | 1125 | 1136 | + | gaaataaacatt |
| MA0031.1 | FOXD1 | 9.41226 | 0.895822 | VEGFA | 1128 | 1135 | + | ataaacat |
| MA0847.1 | FOXD2 | 10.6003 | 0.977827 | VEGFA | 1128 | 1134 | + | ataaaca |
| MA0613.1 | FOXG1 | 12.7432 | 0.975466 | VEGFA | 1128 | 1135 | + | ataaacat |
| MA0479.1 | FOXH1 | 9.82447 | 0.910832 | VEGFA | 1124 | 1134 | + | ggaaataaaca |
| MA0042.1 | FOXI1 | 13.5823 | 0.96368 | VEGFA | 847 | 858 | + | gtttatttgttt |
| MA1103.1 | FOXK2 | 11.1752 | 0.92016 | VEGFA | 1126 | 1136 | + | aaataaacatt |
| MA0033.2 | FOXL1 | 11.9193 | 0.993649 | VEGFA | 1128 | 1134 | + | ataaaca |
| MA0157.2 | FOXO3 | 9.65581 | 0.901758 | VEGFA | 1128 | 1135 | + | ataaacat |
| MA0850.1 | FOXP3 | 10.4458 | 0.9785 | VEGFA | 1128 | 1134 | + | ataaaca |

**Table S2. shRNA Sequences**

| Targeting RNA | Stem Sequence |
| --- | --- |
| CXCR2-LV3-shRNA#1  **CXCR2-LV3-shRNA#2**  CXCR2-LV3-shRNA#3 | CTCATTAGGATGGCTAGTATC  **CCGTCTACTCATCCAATGTTA**  TCCTCAAGATTCTAGCTATAC |
| **FOXD1-LV3-shRNA#1**  FOXD1-LV3-shRNA#2  FOXD1-LV3-shRNA#3 | **CGTATATCGCGCTCATCACTA**  GAACTTTACTGCTAGGATTTC  TTGTTAATAACGCTATGTTAG |

***Bold fonts represent the most effective sequence**

**Table S3. Primer Sequences**

| **Primer name** | **Forward primer** | **Reverse primer** |
| --- | --- | --- |
| FOXD1 | CCGGCTCCTTTTCTCGTCTT | GGGCTGTTGACAGTTTTGTCC |
| FOXA1 | GCTACTACGCAGACACGCAG | GTTGCCGCTCGTAGTCATGG |
| FOXI1 | AAAGGGAATTACTGGACCCTGG | AGGCTGTGCTAGAGGAAACATC |
| FOXL1 | CTCGCCCATGCTGTATCTGT | TGAGCGCGATGTAGCTGTAG |
| FOXO3 | GCCACCCTTGGCCTCTAAAT | AGATCCCAAGAGCGCATGTC |
| FOXK2 | CCGTACAGCCACACATCTCG | CCTTGTACCCTGAAGACCCC |
| FOXP3 | TCCAGGACAGGCCACATTTC | TTGAGGGAGAAGACCCCAGT |
| FOXG1 | GCCAAGTTTTACGACGGGAC | AAGGGTTGGAAGAAGACCCC |
| FOXD2 | TCCCTGAGTCCTCCCGC | CCCGCGTCCGTCTTGAG |
| FOXC1 | CGGGAGATGTTCGAGTCACAG | GGTACAGAGACTGGCTGGAAG |
| FOXC2 | TGTTCGAGAACGGCAGCTT | CGCTCTTGATCACCACCTTCT |
| FOXH1 | CCCCCTCTTGTTCCTAGGGT | CCTGAACTATGGGGGCTGGA |
|  |  |  |
| VEGF-A | AGGGCAGAATCATCACGAAGT | AGGGTCTCGATTGGATGGCA |
| GAPDH | GGACCTGACCTGCCGTCTAG | GTAGCCCAGGATGCCCTTGA |

**Table S4. Primer Sequences for ChIP assay**

| **Primer name** | **Forward primer** | **Reverse primer** |
| --- | --- | --- |
| VEGF-A-1 | CTTCCCTTCCATATCCCGTTCATC | CAACCCTCCCTTTCCATCATTCG |
| VEGF-A-2 | CTTCCCTTCCATATCCCGTTCATC | CCCTCCCTTTCCATCATTCGTG |
| VEGF-A-3 | CCCTTCCATATCCCGTTCATCAG | CCCTCCCTTTCCATCATTCGTG |
