# Supplementary material for: CXCL5 induces tumor angiogenesis via enhancing the expression of FOXD1 mediated by the AKT/NF-κB pathway in colorectal cancer
Source: Cell Death Dis. 2019 Feb 21;10(3):178. doi: 10.1038/s41419-019-1431-6 (PMC6385313; doi:10.1038/s41419-019-1431-6)
Supplement: Supplementary file 5 — Supplemental figure legends [file 41419_2019_1431_MOESM5_ESM.docx]

**Supplementary figure legends**

**Figure S1.** (A) VEGF-A protein level in HUVEC culture media was determined using ELISA after cells were stimulated with 10 ng/ml rhCXCL5 for various times. (B) VEGF-A protein level in HUVEC culture media was determined using ELISA after cells were stimulated with rhCXCL5 in 36 hours with different concentration. (C) VEGF mRNA expression in HUVECs was determined using qPCR after cells were stimulated with 10 ng/ml rhCXCL5 for various times. (D) VEGF mRNA expression in HUVECs was determined using qPCR after cells were stimulated with rhCXCL5 in 36 hours with different concentration. (E) The expression of CXCR2 after downregulation in endothelial cells were determined using western blot. (F) CCK8 assay in different groups. rhCXCL5 promotes HUVECs proliferation ability which can be inhibited by CXCR2 silencing. Data represent the mean ± SD, *P＜0.05, **P＜0.01, ***P＜0.001.

**Figure S2.** (A) The expression of FOXD1 after downregulation in endothelial cells were determined using western blot. (B) CCK8 assay in different groups. HUVECs proliferation ability are reduced by knocking down FOXD1. Data represent the mean ± SD, *P＜0.05, **P＜0.01, ***P＜0.001.

**Figure S3.** (A and B) ChIP-qPCR assay using Flag antibody or control IgG in HUVECs transfected with a FOXD1 (Flag-tagged) plasmid shows the binding of FOXD1 on the VEGF-A promoter. Data represent the mean ± SD, *P＜0.05, **P＜0.01, ***P＜0.001.
